# Weight changes before and after lurasidone treatment: a real-world analysis using electronic health records

**DOI:** 10.1186/s12991-017-0159-x

**Published:** 2017-10-17

**Authors:** Jonathan M. Meyer, Daisy S. Ng-Mak, Chien-Chia Chuang, Krithika Rajagopalan, Antony Loebel

**Affiliations:** 1 Department of Psychiatry, University of California, San Diego, California USA; 2 Sunovion Pharmaceuticals Inc., 84 Waterford Drive, Marlborough, MA 01752 USA; 3 Vertex Pharmaceuticals, Cambridge, MA USA; 4 Sunovion Pharmaceuticals Inc., Fort Lee, NJ USA

**Keywords:** Weight change, Mental illness, Electronic health records, Lurasidone, Antipsychotic treatment

## Abstract

**Background:**

Severe and persistent mental illnesses, such as schizophrenia and bipolar disorder, are associated with increased risk of obesity compared to the general population. While the association of lurasidone and lower risk of weight gain has been established in short and longer-term clinical trial settings, information about lurasidone’s association with weight gain in usual clinical care is limited. This analysis of usual clinical care evaluated weight changes associated with lurasidone treatment in patients with schizophrenia or bipolar disorder.

**Methods:**

A retrospective, longitudinal analysis was conducted using de-identified electronic health records from the Humedica database for patients who initiated lurasidone monotherapy between February 2011 and November 2013. Weight data were analyzed using longitudinal mixed-effects models to estimate the impact of lurasidone on patient weight trajectories over time. Patients’ weight data (kg) were tracked for 12-months prior to and up to 12-months following lurasidone initiation. Stratified analyses were conducted based on prior use of second-generation antipsychotics with medium/high risk (clozapine, olanzapine, quetiapine, or risperidone) versus low risk (aripiprazole, ziprasidone, first-generation antipsychotics, or no prior antipsychotics) for weight gain.

**Results:**

Among the 439 included patients, the mean age was 42.2 years, and 69.7% were female. The average duration of lurasidone treatment across all patients was 55.2 days and follow-up duration after the index date was 225.1 days. The estimated impact of lurasidone on weight was − 0.77 kg at the end of the 1-year follow-up. Patients who had received a prior second-generation antipsychotic with medium/high risk for weight gain were estimated to lose an average of 1.68 kg at the end of the 1-year follow-up.

**Conclusions:**

Lurasidone was associated with a reduction in weight at 1 year following its initiation in patients with schizophrenia or bipolar disorder. Stratified analyses indicated that weight reduction was more pronounced among patients who had received second-generation antipsychotics associated with a higher risk of weight gain prior to lurasidone treatment. These findings are consistent with the results of prior short- and long-term prospective studies and suggest that lurasidone is associated with low risk for weight gain in patients with schizophrenia or bipolar disorder.

**Electronic supplementary material:**

The online version of this article (doi:10.1186/s12991-017-0159-x) contains supplementary material, which is available to authorized users.

## Introduction

Severe and persistent mental illnesses, such as schizophrenia and bipolar disorder, are associated with increased risk of obesity [1] compared to the general population. While over one-third of adults in the United States are obese [body mass index (BMI) ≥ 30 kg/m^2^], [2, 3] the prevalence of obesity or being overweight is estimated at 40–63% among patients with schizophrenia, [4–6] and 49–68% among patients with bipolar disorder [7–10]. Evidence suggests that the presence of obesity using BMI, or central adiposity by waist circumference criteria, may negatively impact the disease course in schizophrenia and bipolar disorder, and increase risks of cardiovascular disease, diabetes, and stroke in addition to reducing health-related quality of life [6, 11].

One of the contributing factors for this higher risk of overweight or obesity in patients with schizophrenia or bipolar disorder may be the use of antipsychotics. While antipsychotics are the current standard pharmacological treatment for severe mental illnesses, as a class they are associated with varying rates of adverse metabolic effects, including a propensity for weight gain [12–15]. Proposed biological mechanisms leading to weight gain in patients treated with antipsychotics include changes in leptin [16] and adiponectin [17]. Among the second-generation antipsychotic (SGA) medications with extensive historical data, clozapine, olanzapine, quetiapine, and risperidone are associated with medium and high risk for weight gain, while aripiprazole and ziprasidone have a lower risk [18, 19]. Lurasidone is an SGA associated with lower risk for inducing weight gain and other cardiometabolic abnormalities in controlled trials and long-term extension studies [15, 20–23]. In addition to the metabolic and social burden of obesity related to SGA use, patients consider the issue of weight gain as the most important treatment concern for the management of bipolar depression [24]. Weight gain is one of the primary adverse effects that lead to treatment non-adherence or discontinuation among schizophrenia patients [25–27]. Non-adherence to psychotropic medications has been found to be associated with consequent relapse and hospitalization rates, resulting in greater healthcare resource use and higher healthcare costs [25, 26, 28, 29].

Given these factors, it is important for clinicians to consider SGAs that are not only efficacious but also provide a lower or more acceptable risk of metabolic disturbance in patients with schizophrenia [30–32]. The value of switching to a lower metabolic risk SGA for patients with high metabolic risk has been reported in other studies [30, 33]. Selection of such SGAs may potentially lead to lowered metabolic risk, improved overall health-related quality of life, and reduced healthcare costs [34]. While the association of lurasidone and lower risk of weight gain has been established in short and longer-term clinical trial settings, information about lurasidone’s association with weight gain in usual clinical care is limited. This study sought to assess the effect of lurasidone treatment on body weight among patients with schizophrenia or bipolar disorder in real-world settings.

## Methods

### Study design

This study was a retrospective, longitudinal analysis of de-identified electronic health records. The health records were from the Humedica NorthStar™ database from February 1, 2011 to December 31, 2013.

### Data source

The Humedica database contains detailed medical information from integrated claims, prescription, and practice management data on approximately 30 million individuals across 38 states in the US [35]. The de-identified data are representative of the US population in terms of distributions of age, gender, and geographical region. Humedica partners with the nation’s leading medical groups, integrated delivery networks, and hospital chains to obtain data from their electronic medical records and information technology systems in real time. The data are then normalized, validated, and aggregated to generate a complete and longitudinal view of patient care [35]. The data used in this study were sourced from the Humedica analytics platform. In addition to the inclusion of standard disease diagnosis and procedure details, the Humedica database includes information of direct relevance to this study, including body weight [kilograms (kg)], body mass index [BMI (kg/m^2^)], and prescription drug information obtained from each provider group’s platform. While Humedica standardizes these data to ensure consistency across values (i.e., weight in kg), there is no additional logic or algorithm applied. All data used in this study were de-identified and are compliant with the Health Insurance Portability and Accountability Act (HIPAA) of 1996. The study did not require institutional review board approval.

### Sample selection

Patients were included in this study if they met the following criteria: (a) had at least one prescription for lurasidone between February 1, 2011 and November 30, 2013 (first lurasidone prescription date was defined as the index date), (b) did not have prescriptions for other antipsychotics or mood-stabilizing agents at the time of the first prescription for lurasidone, (c) did not have any lurasidone prescriptions during the 12 month pre-index period, (d) were aged 18 years or older at index, (e) had ≥ 1 weight recorded during the 12 month pre-index period and ≥ 1 weight recorded during the 12 month post-index period, (f) had ≥ 1 medical encounter in the electronic health record system atleast 12 months prior to the index date, and (g) did not receive other first-generation antipsychotics (FGAs) or SGAs during the 12 month post-index period. Patients were followed for 12 months before the first lurasidone prescription (baseline period) and up to 12 months (minimum 30 days) after the index date (follow-up period).

### Study variables

Demographic characteristics consisted of age at index date (first lurasidone prescription date), gender, and geographic region (Midwest, Northeast, South, West). Duration of follow-up (in days) was truncated at 365 days after the index date. Clinical characteristics included whether the patient had been diagnosed with a schizophrenia spectrum or bipolar disorder [see Appendix: Table 2 for the International Classification of Diseases, Ninth Revision, Clinical Modification (ICD-9-CM) codes], presence of selected comorbidities (i.e., major depressive disorder, substance abuse, hypertension, hyperlipidemia, diabetes, chronic obstructive pulmonary disease; see Appendix: Table 2), and the Charlson Comorbidity Index score [36]. In addition, prior use of FGAs or SGAs, and prior use of other medications (i.e., antidepressants and diuretics) were included. Presence of medication was identified based on the drug names in the electronic health records (see Appendix: Table 3). Pre-index clinical characteristics also included the most recent BMI measure prior to the index date and BMI classification (i.e., underweight < 18.5 kg/m^2^, normal weight 18.5–24.9 kg/m^2^, overweight 25.0–29.9 kg/m^2^, and obese ≥ 30 kg/m^2^). Characteristics of lurasidone treatment included the duration of treatment in days (defined as the number of days between the first and last prescription for lurasidone during the follow-up period), and the number of lurasidone prescriptions during the follow-up period.

The primary outcome variable for this study was patient weight (kg) during pre-index and follow-up periods. Weight observations were trimmed such that any records with extreme weight values (i.e., less than 25 kg or greater than 200 kg) were dropped. Patients were only allowed one unique weight observation per day. In cases where a patient had multiple weight measures on the same day, the first recorded weight (based on the timestamp) was selected. For patients with multiple height measures, the modal value was selected; if no modal value existed, the median result was selected as it was less affected by outliers. Height observations were trimmed so that any values less than 120 cm or greater than 220 cm were dropped from the analysis. The patient’s BMI was calculated using a uniform height measure; therefore, changes in BMI over time reflected changes in weight.

### Statistical analyses

Longitudinal mixed-effects modeling of patient weight trajectories over time was conducted to estimate: (a) patient weight on the index date, (b) the slope of weight change between the beginning and the end of the baseline period (i.e., up until the index date), and (c) the slope of weight change between the beginning and the end of the follow-up period (i.e., a maximum of 365 days after the index date). In addition to the analysis of the overall patient cohort, stratified analyses were conducted on subsets of the overall patient cohort. The first stratified analysis split patients into those who did or did not receive a SGA during the pre-index period. The second split the sample into those who received a SGA with a medium or high risk for weight gain (i.e., clozapine, olanzapine, quetiapine, and risperidone) versus low risk for weight gain (aripiprazole, ziprasidone, first-generation antipsychotics, or no prior antipsychotics).

## Results

**Fig. 1:**
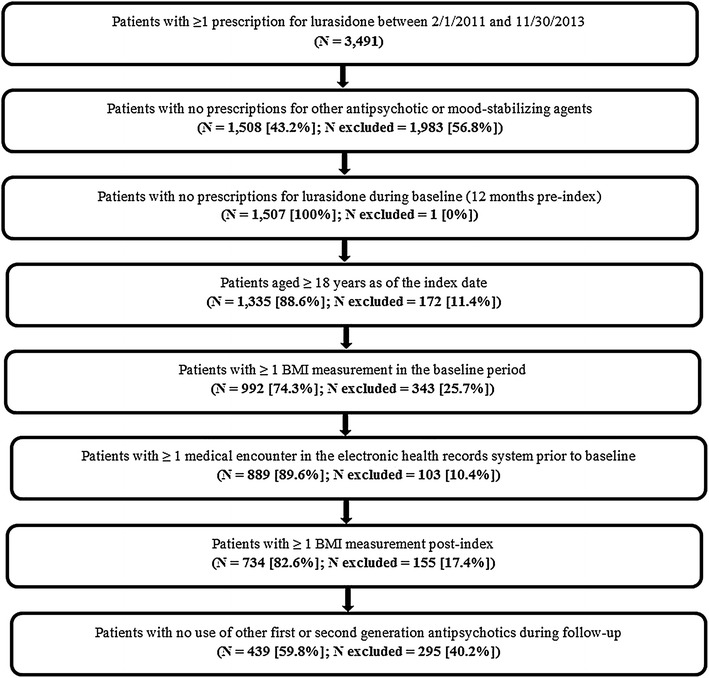
Patient selection flowchart

Of 3491 patients who received at least one lurasidone prescription, 439 patients initiated lurasidone as monotherapy, continued on lurasidone monotherapy, and had multiple weight measures (Fig. 1). Demographics and patient characteristics during the pre-index period are reported in Table 1. The mean sample age was 42.2 years, and 69.7% were female. The mean duration of follow-up after the index date was 225.1 days. At index, 65.8% of patients had a diagnosis of schizophrenia spectrum or bipolar disorder, and 34.2% had other or no diagnoses. Almost half of the patients (45.8%) had prior use of a SGA; of these, a total of 27% of patients had prior use of a SGA associated with medium or high risk for weight gain. First-generation antipsychotics were prescribed to 14.1% of patients in the year prior to lurasidone initiation. Over half of the patients had prior use of antidepressants. Psychiatric and cardiometabolic comorbidities were commonly observed at index. The mean baseline weight for patients included in this analysis was 93.9 kg, and almost two-thirds of patients were classified as obese according to their BMI at index.

**Table 1:** Patient demographic and clinical characteristics at index

Characteristics	Lurasidone (*N* = 439)
Age (years), mean (SD)	42.2 (13.5)
Female, *N* (%)	306 (69.7%)
Follow-up duration (days), mean (SD)	225.1 (124.6)
Diagnosis of schizophrenia or bipolar disorder, *N* (%)	
Schizophrenia spectrum diagnosis	83 (18.9%)
Bipolar disorder diagnosis	206 (46.9%)
Other/unknown diagnosis^a^	150 (34.2%)
Prior use of any second-generation antipsychotics *N* (%)	201 (45.8%)
Prior use of second-generation antipsychotics associated with medium or higher risk for weight gain^b^	119 (27.1%)
Prior use of any first-generation antipsychotic, *N* (%)	62 (14.1%)
Prior use of other medications, *N* (%)	
Antidepressants	246 (56.0%)
Diuretics	90 (20.5%)
Comorbidities, *N* (%)	
Depression	196 (44.6%)
Substance abuse	166 (37.8%)
Hypertension	158 (36.0%)
Hyperlipidemia	155 (35.3%)
Diabetes	89 (20.3%)
Chronic obstructive pulmonary disease	85 (19.4%)
Charlson Comorbidity Index Score, mean (SD)	0.8 (1.3)
Weight^c^ and BMI classification^d^	
Weight (kg), mean (SD)	93.9 (25.4)
Underweight, *N* (%)	4 (0.9%)
Normal, *N* (%)	70 (15.9%)
Overweight, *N* (%)	89 (20.3%)
Obese, *N* (%)	276 (62.9%)

^a^As lurasidone would not be prescribed for any other condition except schizophrenia and bipolar, and the lack of a diagnosis in administrative data does not indicate the lack of the disorder, the data from these patients was deemed acceptable to use in the analysis

^b^Second-generation antipsychotics associated with medium or higher risk of weight gain included clozapine, olanzapine, quetiapine, and risperidone

^c^Weight was the most recent weight observation at index (i.e., weight observation closest to and before index date)

^d^BMI classification based on the closest BMI reading prior to the index date. Underweight, BMI < 18.5 kg/m^2^; normal weight, BMI ≥ 18.5 and < 25.0 kg/m^2^; overweight, BMI ≥ 25.0 and < 30 kg/m^2^; obese, BMI ≥ 30 kg/m^2^

**Fig. 2:**
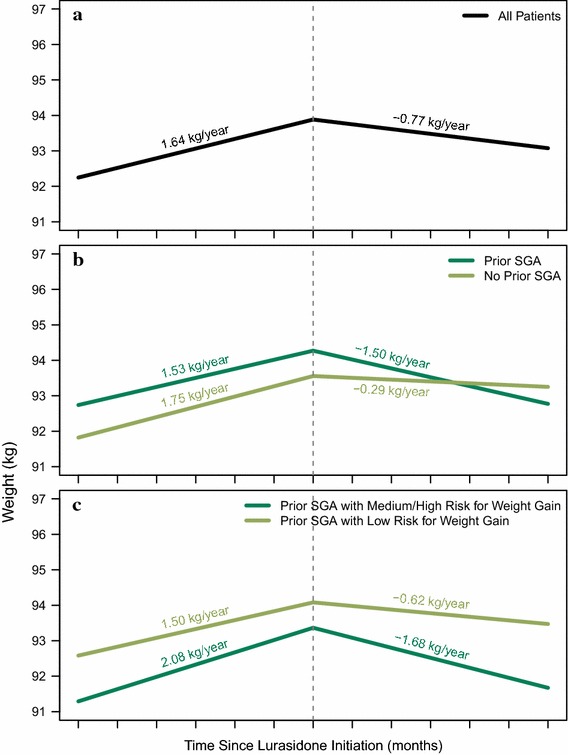
Impact of lurasidone on weight (**a**) overall, and stratified by prior use of second-generation antipsychotics (**b**), and prior use of second-generation antipsychotics with medium/high risk for weight gain (**c**)

The average duration of lurasidone treatment across all patients was 55.2 days, and the majority of patients (76.1%) received lurasidone for < 90 days. On average, patients received two lurasidone prescriptions during the study follow-up period. In the year prior to initiating lurasidone treatment, weight increased by a mean value of 1.64 kg. This trend was reversed after lurasidone initiation (the index date), with patients losing an average of 0.77 kg (Fig. 2a).

Stratified analyses are shown in Fig. 2b, and c. Similar to the overall findings, increases in weight prior to lurasidone initiation and reduction in weight after lurasidone use were observed in all subgroups. The average weight change after lurasidone treatment in subgroups with or without prior use of second-generation antipsychotics were − 1.50 and − 0.29 kg, respectively. The average weight changes after lurasidone treatment in the medium/high weight gain risk antipsychotic subgroups and low weight gain risk antipsychotic subgroups were − 1.68 and − 0.62 kg, respectively (Fig. 2c).

## Discussion

This retrospective, longitudinal study using electronic health records from the Humedica database for patients who initiated lurasidone monotherapy between February 2011 and November 2013 examined patterns of weight change before and after initiating lurasidone therapy in patients with schizophrenia or bipolar disorder. Our findings indicated that lurasidone was associated with a reduction in weight, with an estimated average weight loss of 0.77 kg during the 12-month follow-up period. Weight reduction associated with lurasidone was more pronounced in a subgroup of patients who had previously received SGAs with medium or high risk for weight gain.

Prior switch trials of antipsychotics among patients who changed from an antipsychotic with a higher weight gain risk to one with lower risk have reported that weight loss occurs gradually following the medication change [33, 37]. Six-month open-label extension trials of lurasidone have demonstrated that the weight gained during 6 weeks of exposure to olanzapine can be reversed upon a switch to lurasidone, with the weight loss occurring over 3–4 months [38]. A previous analysis evaluated the effect of 12 months’ treatment with lurasidone on weight in schizophrenia patients using pooled data from eight clinical trials [13]. Meyer et al. reported that weight loss of at least 7% was observed in more than twice as many patients receiving lurasidone (18.5%) than patients receiving risperidone (6.7%) or quetiapine extended release (XR) (9.1%). In addition, patients receiving lurasidone for 12 months were more likely to experience a shift from a higher BMI category to a lower BMI category compared with patients treated with risperidone or quetiapine XR [13]. The results of the current study confirm and extend these prior findings by demonstrating weight loss in real-world patients with schizophrenia or bipolar disorder who were switched to lurasidone for up to 12 months.

Patients with serious mental illness have a greater risk for obesity and associated cardiovascular and metabolic diseases [6, 39]. Antipsychotic-induced weight gain may exacerbate these conditions and lead to treatment discontinuations [25, 26]. The results of this study suggest that a potential strategy to manage antipsychotic weight gain may be to switch patients to an antipsychotic with a lower risk of weight gain, such as lurasidone. In this electronic medical record analysis, patients previously treated with SGAs associated with medium/high risk for weight gain had an average weight loss of 1.68 kg within the 12 months following initiation of lurasidone. Similarly, in a lurasidone switch trial for patients with schizophrenia or schizoaffective disorder, patients lost an average of 0.2 kg at 6 weeks, and more patients had a clinically significant (≥ 7%) weight loss (1.8%) than weight gain (0.9%) [40]. After 6 months of treatment, the average weight loss was 0.8 kg, with 11.8% having clinically significant weight loss. Additionally, patients previously treated with a medium/high risk SGA for weight gain [olanzapine (23.1%) quetiapine (16.7%), and risperidone (22.2%)] appeared more likely to have a clinically significant weight loss than patients who were previously treated with a low risk SGA [aripiprazole (3.2%) or ziprasidone (16.7%)] [41]. Consistent with these findings, a recent meta-analysis of 15 different antipsychotics in patients with schizophrenia, reported significantly less weight gain in patients treated with lurasidone compared with the majority of treatments [19].

Even a relatively modest degree of weight loss (5%) has been associated with metabolic benefits [42] and savings in healthcare costs [43]. This suggests that the weight loss observed for lurasidone-treated patients in this study was meaningful from both clinical and cost perspectives. Pairing the medication switch with a behavioral intervention may lead to an even greater weight loss [44].

The study methodology does have some limitations. The data were collected retrospectively, and other interventions for weight loss could have been implemented by patients at the same time that lurasidone was initiated. Thus, the associations between lurasidone and the reduction in weight following lurasidone initiation could also be due (in whole or in part) to other unobserved interventions such as dietary counseling or prescribed exercise. Mean lurasidone use was only 55.2 days, and weight changes associated with lurasidone were estimated at 1 year. The study was a single-group pre-post assessment and did not include a control group. Therefore, no conclusion could be drawn regarding the relative effectiveness of lurasidone for weight management compared to other antipsychotics or add-on treatments like metformin [45]. Only patients with sufficient weight measurements recorded before and after lurasidone initiation were included in this study. Therefore, this study may have been subject to selection bias towards an overweight/obese cohort whose clinicians were concerned enough to be more assiduous about monitoring weight. It is unclear whether our findings are generalizable to patients with low/normal baseline BMI [46] or to patients who did not gain weight prior to lurasidone treatment. No assessment of cardiometabolic risk factors (e.g., waist circumference or metabolic parameters) were obtained in this study. In addition, assessments of symptomatic change were not available in this study. However, lurasidone is a well-established antipsychotic treatment in patients with schizophrenia and bipolar depression [20, 22–24, 41, 47]. Although one-third of patients in this study did not have a diagnosis code, given the indications for prescribing lurasidone, it is likely that most patients in this study were being treated for schizophrenia spectrum or bipolar disorder.

Another limitation of this study was the linear weight change assumption over a 1-year follow-up period. Prior research suggests that weight change appears to be more pronounced early in treatment and levels off as time passes [38, 48, 49]. To assess the degree to which the choice of a linear model may have resulted in an overestimate of the amount of weight change, a sensitivity analysis (see Additional file 1: Appendix S1) was conducted using 6-month lurasidone data from an open-label extension study [38]. The sensitivity analysis modeled a linear longitudinal mixed-effects model and a nonlinear longitudinal mixed-effects model to test the difference in weight loss estimates. Had a nonlinear model been used in the current study, the weight change estimates may have been about two-thirds the size (see Additional file 1: Appendix S1 for details). While the weight loss estimates may differ somewhat based on whether the weight loss was linear or nonlinear, our study results suggest that lurasidone treatment resulted in statistically significant and clinically meaningful reduction in weight among patients with schizophrenia and bipolar disorder in real-world settings.

## Conclusions

This retrospective, longitudinal study using electronic health records obtained from real-world treatment settings suggests that lurasidone was associated with a reduction in weight at 1 year following its initiation in patients with serious mental illness, primarily schizophrenia or bipolar disorder. Weight reduction was more pronounced among patients who had received second-generation antipsychotics associated with a higher risk of weight gain prior to lurasidone treatment. These findings are consistent with the results of prior short- and long-term prospective studies and suggest that lurasidone is associated with low risk for weight gain in patients with schizophrenia or bipolar disorder.

## Appendix

See Tables 2, 3.

**Table 2:** ICD-9-CM codes used to classify mental and somatic disorders

Comorbidities	ICD-9-CM codes
Schizophrenia	295.XX
Bipolar disorder	296.01, 296.02, 296.03, 296.04, 296.05, 296.06, 296.40, 296.41, 296.42, 296.43, 296.44, 296.45, 296.46, 296.50, 296.51, 296.52, 296.53, 296.54, 296.55, 296.56, 296.60, 296.61, 296.62, 296.63, 296.64, 296.65, 296.66, 296.7, 296.80, 296.89
Major depressive disorder	296.2X, 296.3X, 311.XX
Substance abuse	296.5x, 303.xx–305.xx
Hypertension	401–405
Hyperlipidemia	272.2–272.4
Diabetes	250.XX
Chronic obstructive pulmonary disease	490–496, 500–505, 506.4

*ICD-9-CM* The International Classification of Diseases, Ninth Revision, Clinical Modification. ICD-9-CM was the diagnostic coding available in administrative data during the period of this study

**Table 3:** Medications used in database query

Drug name	Drug name
First-generation antipsychotics	Chlorpromazine, fluphenazine, haloperidol, loxapine, perphenazine, pimozide, thiothixene, thioridazine, and trifluoperazine
Second-generation antipsychotics	Clozapine, olanzapine, quetiapine, risperidone, paliperidone, aripiprazole, ziprasidone, iloperidone, and asenapine
Second-generation antipsychotics that placed patients at higher risk of weight gain	Clozapine, olanzapine, quetiapine, risperidone
Antidepressants	
Diuretics	

## Additional file

**Additional file 1.** Analysis examining potential overestimation of weight change by linear model.

## Abbreviations

BMI: body mass index
cm: centimeter
FGA: first-generation antipsychotic
HIPAA: Health Insurance Portability and Accountability Act
ICD-9-CM: International Classification of Diseases, Ninth Revision, Clinical Modification
kg: kilogram
SGA: second-generation antipsychotic
XR: extended release
