# Supplementary material for: Weight changes before and after lurasidone treatment: a real-world analysis using electronic health records
Source: Ann Gen Psychiatry. 2017 Oct 17;16:36. doi: 10.1186/s12991-017-0159-x (PMC5646018; doi:10.1186/s12991-017-0159-x)
Supplement: Supplementary file 1 — Additional file 1. Analysis examining potential overestimation of weight change by linear model. [file 12991_2017_159_MOESM1_ESM.docx]

**Appendix S1**

Analysis Examining Potential Overestimation of Weight Change by Linear Model

**Background**

The statistician George Box eloquently stated, "Essentially, all models are wrong, but some are useful" (Box & Draper, 1987). His statement points out that all statistical models are, by definition, a simplification of reality, while highlighting that the value of a model is in providing a meaningful understanding and accurate predictions of the phenomenon being studied. This idea, combined with the principal of parsimony, drove the original decision to fit the weight change over time as a linear model.

Treatment-emergent weight loss cannot continue to decrease in a linear fashion over time or individuals would eventually have negative body weight. Positive linear change presents the same issue, with weight eventually growing outside what is reasonable. From a logical perspective, long-term weight change, in reaction to an intervention, must either oscillate over time between reasonable bounds or asymptote to a fixed level. An examination of the form of antipsychotic induced weight change over time from prior studies reveals a form that appears to initially change quickly and then to level off (Bushe et al., 2012, Millen et al. 2011, Meyer et al. 2015, Stahl et al, 2013). Figure 2 from the Stahl et al. (2013) study is reproduced below to demonstrate.

An intrinsically nonlinear model can capture the form of change that occurs quickly at first, and then asymptotes. One model that fits this criteria is weight change = δ × (1 – exp(-α × time)), which has two parameters, δ which represents the amount of weight change to asymptote, and α which must be positive and represents the rate of change that can be converted to a half-life with the formula ln(2)/α (Willet, 1988, Grimm et al., 2011). Supplemental Figure 2 provides a graphical depiction of this model for weight change loss of 3 kilograms (kg) with a half-life of 25 days. The model assumes a fixed half-life, meaning that amount of change will be halved in each period of the specified length. From a clinical perspective, this model answers two interesting questions: (1) which treatment leads to more change (δ) and (2) which treatment leads to a faster change (α).

While weight change in the short-term can be informatively modeled using a simple linear function, if the underlying function is nonlinear, the linear model may overestimate the amount of weight change or loss. To examine the potential limitations of modeling weight change with a simple linear function, both a linear longitudinal mixed effects model and a nonlinear mixed effects model were fit to the weight change data from a randomized clinical trial (Stahl et al., 2013). The predictions at the end of the study were compared.

**Methods**

In the Stahl et al. (2013) trial, 478 patients with schizophrenia were randomized to lurasidone, placebo, or olanzapine for 6 weeks after which time 254 patient entered into a 6-month open label lurasidone treatment extension study. In this exploratory analysis, linear and nonlinear mixed effects models were fit to weight change during the period patients were treated with lurasidone. The first set of models was fit for those randomized to lurasidone and this period ran from randomization to the end of the open label extension study (6 weeks plus 6 months). The second set of models was fit for those initially randomized to olanzapine or placebo this period included only the open label lurasidone extension study (6 months). In this second set of models, the prior treatment was coded 0 for placebo and 1 for olanzapine. See Stahl et al (2013) for further details of the design of the study used for this exploratory analysis.

All models were fit in R version 3.30 (R Core Team, Vienna, Austria) using the Linear and Nonlinear Mixed Effects Models (nlme) package (version 3.1-127). For each model, all of the parameters were included as both a fixed and random effect.

**Results**

For the exploratory analysis of the Stahl et al. (2013) data, linear and nonlinear mixed effects models were fit for (1) participants randomized to lurasidone for the first 6 weeks and across the 6-month extension and (2) for participants initially randomized to placebo or olanzapine and switched to lurasidone for the 6-month extension study. Supplementary Table 1 gives the estimates and significance tests for these models.

The first set of models, fit to patients initially treated with lurasidone, showed a predicted weight gain at the end of the full study (day 225) of 3.21kg for the linear model (ie, 0.34439 + 0.01273 × 225= 3.21) and 1.58kg for the nonlinear model (ie, 1.583 × (1 – exp(-0.028 × 225 days)). In the second set of models, patients who were initially treated with placebo were estimated to gain 1.13kg after 180 days of lurasidone treatment by the linear model and 0.95kg by the nonlinear model. The patients, who were initially treated with olanzapine, were estimated to lose 3.13kg by the linear model and 2.27 kg by the nonlinear model after 180 days of lurasidone treatment. Supplemental Figure 3 gives the predicted values for the different models overtime along with the observed means at each treatment visit.

**Discussion**

To assess the degree to which the methodology may have overestimated the amount of weight change, we modeled data from another study (Stahl et al., 2013) using both a linear longitudinal mixed effects model, that was similar to the model used in the primary analysis, and a nonlinear longitudinal mixed effects model. Relative to the linear models, the nonlinear models estimated a lower magnitude of weight change over 7.5 months for those initially randomized to lurasidone (1.58kg/3.21kg = 0.49), for those initially randomized to placebo for 6 weeks and switched to lurasidone for 6 months (0.95kg/1.13kg = 0.84), and for those initially randomized to olanzapine and switched to lurasidone for 6 months (-2.27kg/-3.13kg = 0.72). Had a nonlinear model been used in the current study, the weight change estimates may have been about two-thirds the size.

Antipsychotic-emergent weight gain has found to be greatest with olanzapine and clozapine (Muench and Hamer, 2010). There was a significant difference in weight change for patients who were switched from olanzapine or placebo to open label treatment with lurasidone. While patients switched from placebo to lurasidone gained a small, but statistically significant amount of weight (0.95kg) over 6 months, those who switched from olanzapine lost 2.27kg in the same time period. Lurasidone may be a good next option to switch a patient who discontinues an antipsychotic due to antipsychotic-emergent weight gain.

The nonlinear model use to fit the data in this analysis is probably not the “true” underlying function that describes antipsychotic treatment emergent weight gain over time. However, this model appears to be an informative model that suggests both an overall amount of change, and that the half-life for change is approximately 24 days. While both the linear and nonlinear models provided meaningful information about the direction and magnitude of weight change, the nonlinear model estimated less weight change at the end of the study apparently due to the difference in functional form.

Table S1. Estimates from the linear and non-linear mixed effects models.

|  | Estimate | S.E. Estimate | P |  |
| --- | --- | --- | --- | --- |
| Lurasidone over full Study | | | | |
| Linear |  |  |  |  |
| b_0_ | 0.344 | 0.0953 | <0.001 |  |
| b_1_ | 0.013 | 0.0032 | <0.001 |  |
| Nonlinear |  |  |  |  |
| δ | 1.583 | 0.2727 | <0.001 |  |
| α | 0.028^a^ | 0.0016 | <0.001 |  |
| Lurasidone during Extension Following Placebo or Olanzapine | | | | |
| Linear |  |  |  |  |
| b_0_ | 0.371 | 0.2857 | 0.194 |  |
| b_1_ | 0.004 | 0.0048 | 0.381 |  |
| b_0_*Olanzapine | -0.795 | 0.3922 | 0.045 |  |
| b_1_*Olanzapine | -0.019 | 0.0066 | 0.004 |  |
| Nonlinear |  |  |  |  |
| δ | 0.956 | 0.6345 | 0.133 |  |
| α | 0.029^b^ | 0.0063 | <0.001 |  |
| δ*Olanzapine | -3.262 | 0.8733 | <0.001 |  |
| α*Olanzapine | -0.006^c^ | 0.0065 | 0.386 |  |

b_0_ – intercept in linear model

b_1_ – slope per day in linear model

^a^ Half-life of 24.0 days.

^b^ Half-life of 24.7 days.

^c^ Half-life of 29.8 days.

**Additional References**

Box, G.E.P., Draper, N.R., 1987. Empirical Model-Building and Response Surfaces, John Wiley & Sons, Inc. p. 424.

Grimm K.J., Ram N., Hamagami F., 2011. Nonlinear growth curves in developmental research. Child Dev. 82:1357–71.

Willet J.B., 1988. Questions and answers in the measurement of change, in: Rothkopf, E. (Ed.), Reviews of Research in Education. American Education Research Association, Washington, DC, pp. 345-422.

**Figure S1. Weight Change over Time from Stahl et al., 2013.**


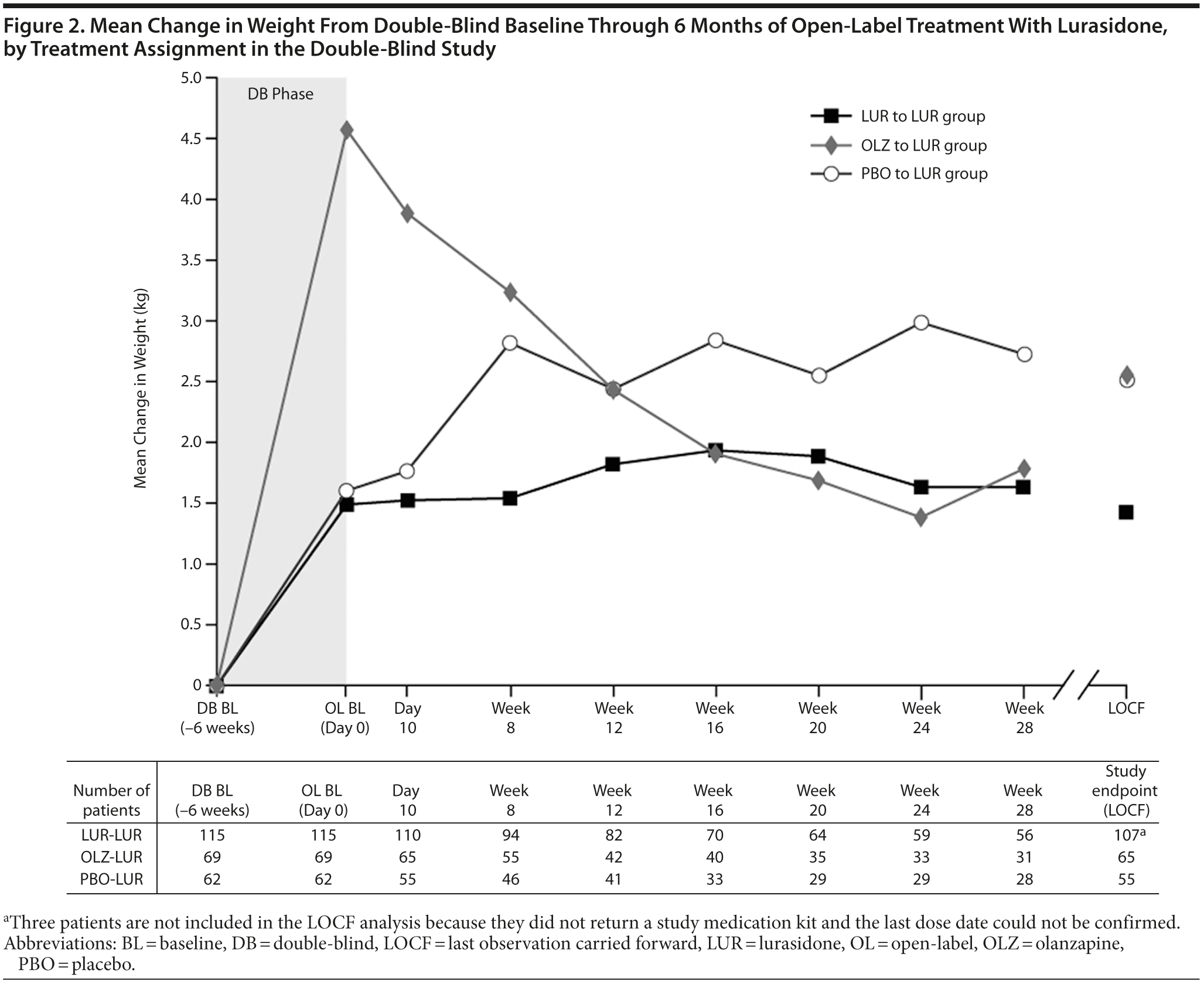


Available open access at (http://www.psychiatrist.com/jcp/article/Pages/2013/v74n05/v74n0516.aspx).

**Figure S2. Illustration of Non-linear Model.**

The nonlinear model with δ = -3 kg and the half-life = 25 days (i.e., α = 0.0277 and *ln*(2)/α = 25). The total change is 3 kgs and after 25 days the weight has is half the δ amount. Each period of 25 days leads to an additional change of half the amount from the previous period.

**Figure S3. Model Predictions and Observed Means.**

Points represent the observed means at each visit.
